# Coordinating Care for Falls *via* Emergency Responders: A Feasibility Study of a Brief At-Scene Intervention

**DOI:** 10.3389/fpubh.2016.00266

**Published:** 2016-12-01

**Authors:** Elizabeth A. Phelan, Julia Herbert, Carol Fahrenbruch, Benjamin A. Stubbs, Hendrika Meischke

**Affiliations:** ^1^Division of Gerontology and Geriatric Medicine, Department of Medicine, University of Washington, Seattle, WA, USA; ^2^Department of Health Services, School of Public Health, University of Washington, Seattle, WA, USA; ^3^Medical College of Wisconsin Affiliated Hospitals, Milwaukee, WI, USA; ^4^EMS Division, Public Health – Seattle and King County, Seattle, WA, USA; ^5^Department of Family Medicine, University of Washington, Seattle, WA, USA

**Keywords:** accidental falls, aged, prehospital care, emergency medical technicians, public health, perception, health services for the aged/organization and administration

## Abstract

Falls account for a substantial portion of 9-1-1 calls, but few studies have examined the potential for an emergency medical system role in fall prevention. We tested the feasibility and effectiveness of an emergency medical technician (EMT)-delivered, at-scene intervention to link elders calling 9-1-1 for a fall with a multifactorial fall prevention program in their community. The intervention was conducted in a single fire department in King County, Washington and consisted of a brief public health message about the preventability of falls and written fall prevention program information left at scene. Data sources included 9-1-1 reports, telephone interviews with intervention department fallers and sociodemographically comparable fallers from three other fire departments in the same county, and in-person discussions with intervention department EMTs. Interviews elicited faller recall and perceptions of the intervention, EMT perceptions of intervention feasibility, and resultant referrals. Sixteen percent of all 9-1-1 calls during the intervention period were for falls. The intervention was delivered to 49% of fallers, the majority of whom (75%) were left at scene. Their mean age (*N* = 92) was 80 ± 8 years; 78% were women, 39% had annual incomes under $20K, and 34% lived alone. Thirty-five percent reported that an EMT had discussed falls and fall prevention (vs. 8% of comparison group, *P* < 0.01); 84% reported that the information was useful. Six percent reported having made an appointment with a fall prevention program (vs. 3% of comparison group). EMTs reported that the intervention was worthwhile and did not add substantially to their workload. A brief, at-scene intervention is feasible and acceptable to fallers and EMTs. Although it activates only a small percent to seek out fall prevention programs, the public health impact of this low-cost strategy may be substantial.

## Introduction, Background, and Rationale

Accidental falls occur commonly among older people (1), often cause serious injuries (2, 3), and account for a substantial portion of 9-1-1 calls (4–6). With the growth of the elderly population, this situation is likely to persist or even worsen. Prevention of falls is thus imperative, and system-level strategies to improve identification and management of those at high risk of falls and fall-related injuries are essential. Evidence suggests that emergency medical service (EMS) providers can engage and educate lay persons and affect practice for a number of important health conditions (7), and firefighters and emergency medical technicians (EMTs) are a well-trusted information source. However to-date, few studies have assessed the potential for an EMS role in fall prevention (8, 9), and data on the feasibility and effectiveness of proactive outreach by EMS providers in the context of a 9-1-1 call for a fall are lacking. Because of the widespread availability of EMS services throughout the United States, examination of an active EMS role in the prevention of falls is warranted. We thus sought to assess the feasibility and preliminary effectiveness of an EMS-delivered, brief at-scene intervention describing the preventability of falls and locally available community resources for fall prevention. We used a posttest only, comparison group evaluation design. We hypothesized that fall-related education, fall prevention program referral information, and encouragement from an EMT at scene during a 9-1-1 response to a fall would be remembered and perceived as useful by the 9-1-1 caller and would result in follow-through on the recommended referral. We further hypothesized that EMTs would consider the activity worthwhile and doable within the context of their at-scene work.

## Materials and Methods

### Setting and Participants

The study was conducted in King County, Washington. The intervention targeted adults aged 65 years and older living in a private residence who called 9-1-1 for a fall. Individuals residing in a skilled nursing facility, adult family home, or assisted living facility were not included in the research evaluation, although the intervention may have been carried out with individuals in those settings who called 9-1-1 for fall-related assistance. Individuals who met inclusion criteria but were transported by advanced life support to an emergency department were also excluded from the research evaluation.

### Intervention Content and Implementation

**Figure 1:**
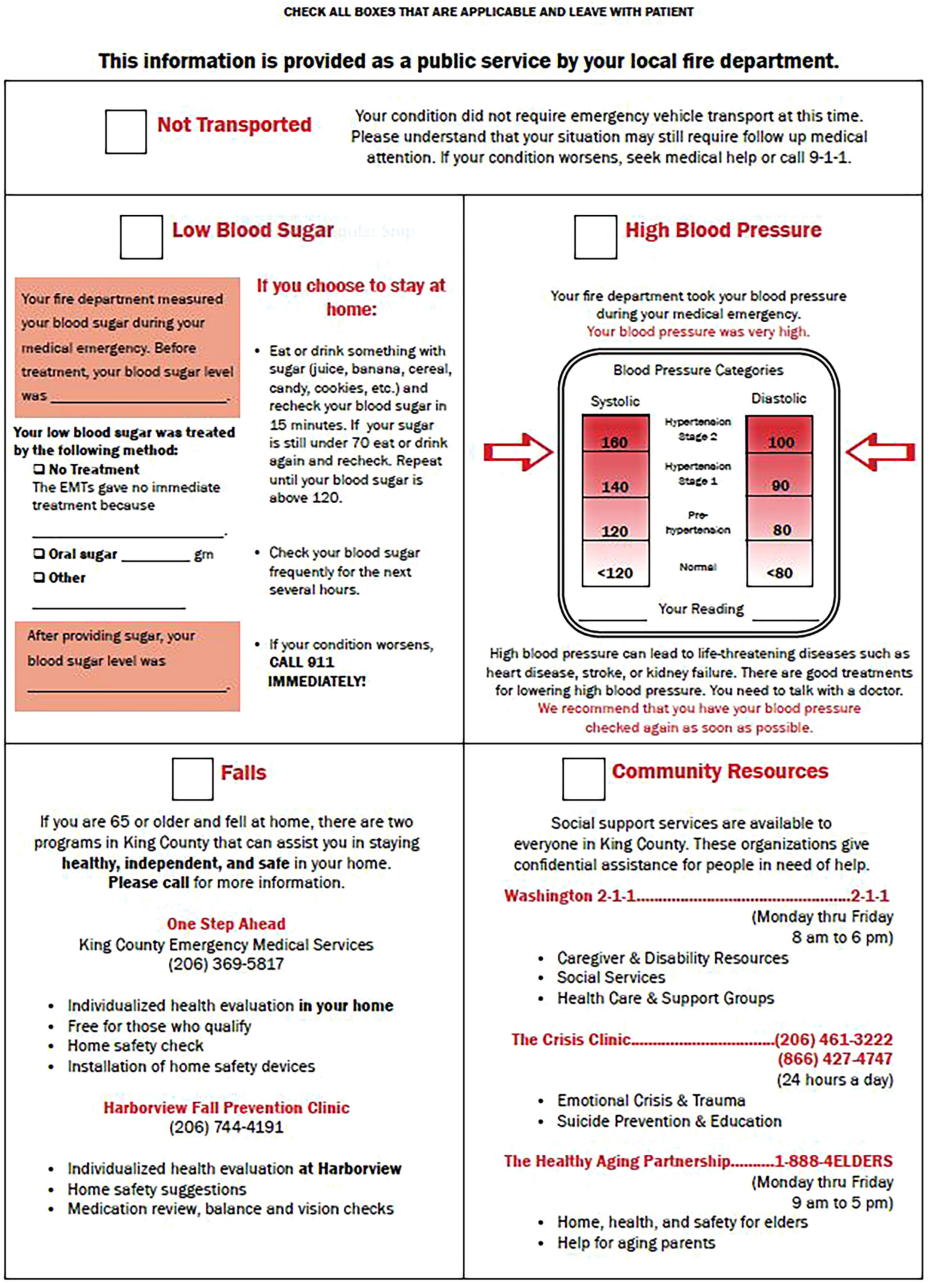
**Medical Incident Report Form (MIRF) tear-off information sheet**. Figure from (10).

The intervention consisted of at-scene counseling by EMTs about the preventability of falls and the availability of local fall prevention programs. A tear-off sheet with information about the locally available programs was developed specifically for the project with input from EMS advisors (Figure 1) (10). One program was a home-based program; the other was a falls assessment clinic operating at the county hospital.

The intervention was implemented by EMTs in the intervention fire department between January 1 and September 30, 2010. The service area of the fire department is roughly 50 mi^2^, with a population of ~140,000. The department’s demographics resemble those of Washington State, with ~10% aged 65 years or older and ~50% females. At the time the study was conducted, there were 6 fire stations and 134 EMTs employed in the department.

Emergency medical technicians underwent a 2-h, in-person training conducted by the project principal investigator and project coordinator 1 month prior to the intervention start period. The training included coaching and role-play in the script covering the serious consequences of falls and their preventability, along with education about the two community fall prevention programs. Two refresher sessions run by the project coordinator were delivered at each fire station in the intervention department in March and July 2010.

## Research Procedures

### Evaluation Design

We used a posttest only, comparison group design for the evaluation. The comparison group sample was drawn from three fire departments within King County, each of which had census-level sociodemographic characteristics comparable to those of the intervention fire department. The number of annual 9-1-1 calls for falls received by the three fire departments is comparable to the number received by the intervention fire department. No standard approach to encouraging falls follow-up care is mandated or followed in any of the three departments, and EMTs may or may not counsel 9-1-1 fallers with regard to the preventability of falls or discuss services available in the community. As this was a feasibility study, formal sample size calculations were not performed (11). The University of Washington Institutional Review Board and the Research Administrative Review Committee, Seattle/King County Public Health Department approved all study procedures.

### Data Sources

Data sources for the evaluation included medical incident report forms (MIRFs) completed at scene by the EMT responding to the 9-1-1 call; informal, in-person discussions with EMT crews in the intervention department, conducted by the project coordinator during month 9 of the intervention period; telephone interviews with fallers in the intervention and comparison departments, conducted by trained research assistants within 1 month of the faller’s 9-1-1 call and after obtaining oral consent for interview participation; and fall prevention program records.

### Recruitment for Telephone Interview Participation

Adults aged 65 years and older residing in a private residence located in either the intervention or comparison fire departments who called 9-1-1 for a fall during the intervention period were potentially eligible for a telephone interview. Name and contact information for these individuals were recorded in an electronic database at the EMS Division’s central office and were accessible only to the project coordinator, an EMS Division employee. Names and phone numbers of fallers potentially eligible for a telephone interview were transmitted by the project coordinator to the research assistant. The research assistant called each potentially eligible person within 1 month after the 9-1-1 incident, confirmed interview eligibility, obtained oral consent, and thereafter conducted the telephone interview. The interview assessed what risk reduction activities the faller had engaged in after his/her 9-1-1 call, beliefs about fall prevention, and personal risk of falls and, for fallers in the intervention department, whether he/she had been referred to and had made an appointment to be evaluated by one of the fall prevention programs. Those who called 9-1-1 more than once for a fall during the intervention period were interviewed only once.

### Fall Prevention Program Referral Follow-through

The research assistant contacted the fall prevention programs each month to determine which of the fallers who were eligible for referral to the fall prevention programs had been scheduled with and/or seen and evaluated by a program specialist.

### Measurement

Feasibility was assessed by the percentage of 9-1-1 calls for falls wherein falls aftercare was provided, as measured by MIRF documentation that the tear-off information sheet was left at scene, by EMT crew perceptions of the intervention and ease of incorporating it into their 9-1-1 runs, and by faller recall and perceptions of the intervention, as measured by telephone survey items. Our primary outcome of interest for the purpose of this feasibility study was the intervention’s effect on getting fallers connected to fall prevention services in their community (i.e., “coordinating care for falls”). Effectiveness was thus measured by the proportion of fallers receiving a formal fall-risk assessment by a trained health professional (regardless of specific fall prevention program option chosen). We also assessed the proportion engaging in evidence-based fall prevention activities as a secondary outcome.

### Statistical Analyses

Descriptive statistics were used to characterize study participants, extent of intervention delivery, and faller recall and perceptions of the intervention. Categorical variable proportions were compared by chi-square tests or by Fisher’s exact test if one or more expected cell frequencies was less than five. Continuous variable means were compared using *t* tests. Two-sided statistical significance was set at *P* ≤ 0.05. All analyses were conducted using the IBM Statistical Package for the Social Sciences (SPSS), version 20.

## Results

### Participant Flow

**Figure 2:**
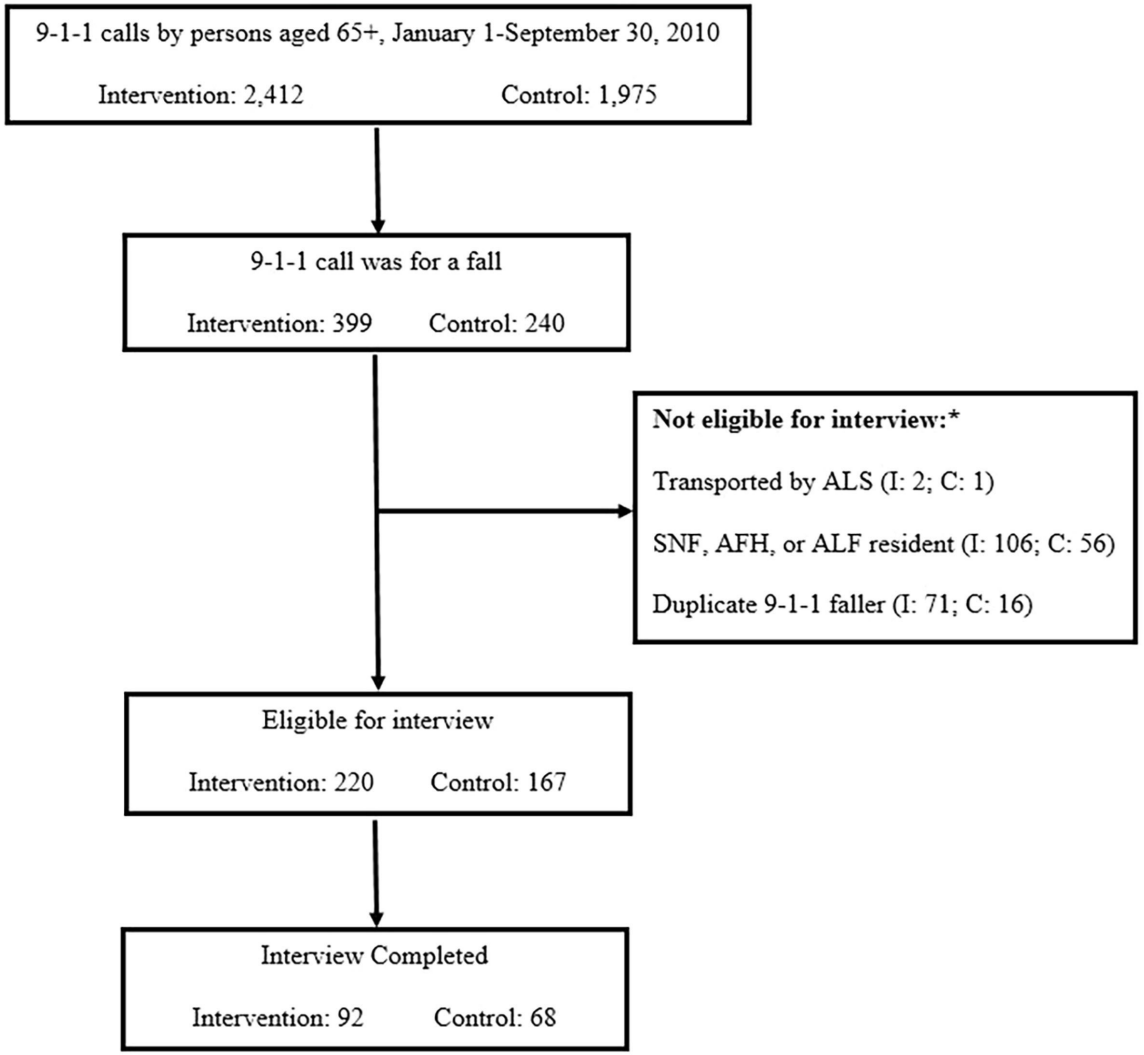
**Participants interviewed *via* telephone from intervention and control fire departments**. *ALS, advanced life support; SNF, skilled nursing facility; AFH, adult family home; ALF, assisted living facility.

Falls accounted for 12–16% of all calls from persons aged 65 years and older in the intervention and comparison fire departments during the intervention period (Figure 2).

Figure 2 shows the number of interviews completed in the intervention and control fire departments among those who met criteria for inclusion in the research evaluation. The most frequent reasons for interview non-completion were inability to locate a valid telephone number despite an in-depth search (27%), declining to be interviewed (14%), and inability to reach despite 10 attempts (8%).

### Participant Characteristics

Table 1 shows characteristics of those interviewed by study group. Both groups were predominantly females with an average age of 80. A substantial proportion were low-income, living alone, and reported fair or poor health. Just over half in each group rated preventing falls as extremely important to their overall health. About one-quarter reported not knowing what their chances were of falling again in the future, and another quarter to one-third believed that they were not at all likely to fall again.

**Table 1:** **Demographic and health characteristics and fall-related beliefs of telephone interview participants**.

Characteristic	Intervention (*N* = 92)	Comparison (*N* = 68)	*P*
Age, years, mean ± SD	80 ± 8	80 ± 9	1.00
Female, %	78	64	0.06
Non-white, %	10	7	0.56
Annual income <$20,000, %	39	29	0.20
Living alone, %	34	29	0.57
Health rated fair or poor, %	33	24	0.21
Preventing falls extremely important, %	57	61	0.63
Likelihood of falling again in future			0.03
Extremely/very, %	22	6	
Somewhat/a little, %	28	35	
Not at all, %	21	33	
Don’t know, %	29	26	

### Feasibility

Forty-nine percent of 9-1-1 calls for falls had documentation that falls aftercare was provided (i.e., checkbox marked on MIRF), and 10% of MIRF narratives had some mention that fall prevention was discussed. Seventy-five percent of these were with fallers who were left at scene.

All EMT crews (*N* = 18) in the intervention fire department participated in discussions to elicit their views on the intervention. EMTs perceived the intervention positively, reporting that it was useful and worthwhile. Representative comments included, “it targets a population in need of attention (many have repeat falls).” They also noted that it did not add significantly to their workload, commenting in fact that, “having a phone number to call from the scene rather than relying on the faller to call a fall prevention program themselves would be useful.” They recognized that the intervention might not be appropriate for people with serious injuries. They also described how they were implementing the intervention – for example, “enlisting a family member is often more helpful than talking only with a faller.”

The vast majority of fallers from both groups recalled their 9-1-1 encounter (Table 2). Significantly more of the intervention group reported that the firefighter had spoken with them about falls and fall prevention. Significantly more also remembered the tear-off sheet of fall prevention program information. A majority of both groups reported that it was useful to have the firefighters talk with them about fall prevention. EMTs were uniformly highly regarded by the 9-1-1 callers, characterized as “prompt,” “kind,” “courteous,” and “caring.”

**Table 2:** **Recall and perceptions of the intervention by telephone interview participants**.

	Intervention (*N* = 92) %	Comparison (*N* = 68) %	*P*
Recall 9-1-1 encounter	94	91	0.88
Firefighter talked about fall prevention	35	8	<0.01
Recall tear-off sheet	6	2	<0.01
Fall prevention discussion useful	84	75	0.17

### Effectiveness

Six percent of the intervention group reported having made an appointment with a fall prevention program (vs. 3% of the comparison group). Multiple reasons for not having done so were cited, with no reason predominating – examples included “being too busy,” “already getting a lot of help,” “working on things on own at home,” and “the fall could have happened to anyone.” However, a majority in both groups (79% intervention, 73% controls) reported that they had made changes to their home or daily activities to prevent themselves from falling again. Table 3 shows the self-care and care-seeking behaviors to reduce the risk of falls reported by interview participants. Referral data from the programs showed that 12 referrals were received from the intervention fire department during the intervention period and 7 from the control fire departments. All referrals were to the home-based fall prevention program.

**Table 3:** **Fall prevention behavior changes reported by telephone interview participants**.

Behavior	Intervention (*N* = 92) %	Comparison (*N* = 68) %	*P*
Evaluated by a health-care provider	1	0	1.00*
Exercising more	5	5	0.77
Changed medications	6	2	0.24*
Added home safety devices	21	10	0.08
Became more careful	25	29	0.53

*Fisher’s exact test.

## Discussion

### Summary of Main Results

This study demonstrated that a brief, at-scene intervention is feasible for EMTs to deliver to community-dwelling older adults who fall and call 9-1-1, particularly among older adults left at scene. With regard to our hypothesis of intervention feasibility, surveys of fallers and discussions with EMT crews suggested that the at-scene intervention was acceptable to both, doable, and worthwhile. During the study period, filling out the checkboxes on the MIRF was voluntary (i.e., not a requirement of the fire department) for EMTs, and our results thus likely underestimate the number of times EMTs provided falls aftercare information during their at-scene encounters. With regard to our hypothesis about effectiveness in prompting fall prevention behavior change, including care-seeking to prevent falls, only a small percent sought out an organized fall prevention program following the intervention, but among those who did, an in-home program was preferred. Other findings worth noting are that the intervention did not appear to influence understanding of one’s personal risk of future falls. In addition, although most reported having made changes to reduce their risk of falls subsequent to their 9-1-1 call, other than home safety modifications, many of those changes have not been well studied and to-date do not have a great deal of evidence behind them.

### Comparison to Other Studies

Prior research has noted the EMS providers are in an opportune position to provide fall-risk-reduction interventions and/or referrals to community programs and services (12). Ours is one of the few tests of an EMT-delivered, at-scene, public-health-oriented outreach intervention to prevent future falls among elder community-dwelling 9-1-1 callers. The frequency of 9-1-1 calls for falls in our study (12–16%) was very consistent with national data showing that among adults aged 65+, calls for falls account for 17% of all EMS calls (12). Older adults who have fallen and called 9-1-1 are at very high risk for recurrent falls (13) and serious injury or death. Given that at least a quarter of those who call 9-1-1 for a fall do not require transport to a health-care facility for emergency care (12, 14), there is enormous opportunity to reach this highly vulnerable group (12, 13) with timely prevention efforts as part of the at-scene EMS response. The intensity of the intervention that achieves the optimal effect in terms of motivating older adult behavior change remains uncertain and is a key area for future study.

### Implications for Community Agencies, Clinicians and Public Health Practitioners, and Research

Community agencies are essential to a comprehensive approach to the delivery of fall prevention services to community-dwelling older adults. Among community agencies, EMS providers are often the first to attend to older adults who have fallen. Efforts to address the issue of frequent, and often-recurrent, 9-1-1 calls for falls (15) are occurring at the grassroots level, led by EMS personnel in multiple communities across the nation. These efforts are typically homegrown, and the interventions often innovative, but evaluation to determine their effects is often insufficient. Partnerships with evaluators could strengthen understanding of any given intervention on key effects such as motivating older adults to take action to prevent future falls.

Because of its relative simplicity, the intervention we developed should be readily adoptable by other EMS systems across the United States. However, the availability of falls clinics and/or other fall prevention programs is limited in many communities, and so local readiness to implement our intervention would first need to be assessed. Furthermore, because EMS programs are typically emergency-services-oriented, EMS leadership must endorse a more preventive role to allow for a shift in the traditional paradigm to occur. Adoption of this new role by EMTs depends on leadership buy-in, encouragement, and change in perceptions of an expanded mission of EMS (10).

From the perspective of public health practice, intensified efforts to raise population awareness of effective fall prevention strategies is crucial, given the predilection of those we studied to take personal action to prevent future falls, independent of organized fall prevention programs.

Researchers interested in conducting pragmatic trials have ample opportunity for design and testing of interventions delivered within the context of 9-1-1 responses. Interventions could focus not only on falls and fall prevention but also on other conditions for which 9-1-1 calls commonly occur. Studies of the efficacy of “being more careful” and other seemingly non-evidence-based fall prevention strategies that older adults in our study pursued are also warranted, since acceptance of generally recommended interventions is low (16).

### Strengths

A key strength of our study is its pragmatic orientation and the community-based research evidence generated. Our data permit realistic estimates of the rate of uptake of available community-based fall prevention resources by older adults when offered. This is in contrast to the data generated in the context of rigid trial circumstances (8, 17, 18) wherein healthy volunteer bias may result in levels of adherence (i.e., follow-through on referrals) that are unlikely to be achieved under real-world conditions. We and others have previously documented low engagement in fall prevention activities (19, 20), and so the importance of such real-world data cannot be underestimated.

### Limitations

This study has several limitations, most of which represent threats to internal validity. The quasi-experimental (non-randomized) evaluation design limits causal inference. In addition, evaluation findings are susceptible to selection bias, as ours was essentially a convenience sample. However, our study groups appeared to be quite comparable, at least with regard to their sociodemographic characteristics. Furthermore, the evaluation relied heavily on data obtained from telephone surveys with older adults, and recall and/or social desirability may have affected responses. However, it is unlikely that recall and/or social desirability would have occurred with differential frequency by study group. Lastly, our study was conducted in a single, predominantly urban county in the Pacific Northwest, which limits generalizability. Additional studies in other settings are thus warranted.

## Conclusion

Emergency medical service-attended falls represent an important case-finding and prevention opportunity. The present study suggests that an EMT-driven approach involving brief counseling at scene and recommendation about local fall prevention programs is well received. A somewhat more intensive intervention – for example, one that facilitates placement of a referral to a fall prevention program in real time, and/or includes communication with the patient’s routine source of primary care – may increase the number of fallers who ultimately receive fall prevention services. Additional studies are needed to address this question and to assess whether an augmented intervention would affect key outcomes, including fall-related 9-1-1 calls and ED visits, fall and fall injury rates, and quality of life.

## Author Contributions

Conception and design of the study: HM and EP. Acquisition of data, analysis, or interpretation of data: CF, JH, BS, HM, and EP. Drafting the article or revising it for important intellectual content, final approval of the version to be published, and accountability for accuracy and integrity of the work: CF, JH, HM, EP, and BS.

## Conflict of Interest Statement

The authors declare that the research was conducted in the absence of any commercial or financial relationships that could be construed as a potential conflict of interest.
